# Genetic polymorphisms and their association with neurobiological and psychological factors in anorexia nervosa: a systematic review

**DOI:** 10.3389/fpsyg.2024.1386233

**Published:** 2024-06-21

**Authors:** Heba Almaghrbi, Hiba Bawadi

**Affiliations:** ^1^Department of Biomedical Science, College of Health Sciences, QU Health, Qatar University, Doha, Qatar; ^2^Department of Human Nutrition, College of Health Sciences, QU Health, Qatar University, Doha, Qatar

**Keywords:** anorexia nervosa, eating disorder, genetic polymorphism, genetic susceptibility, neurobiological factors, psychological factors

## Abstract

**Background and aims:**

Anorexia nervosa (AN) is a complex neuropsychiatric disorder. This systematic review synthesizes evidence from diverse studies to assess and investigate the association between gene polymorphisms and psychological and neurobiological factors in patients with AN.

**Methods:**

A systematic search across PubMed, PsycINFO, Scopus, and Web of Science databases, along with manual searching, was conducted. The review protocol was approved by PROSPERO (CRD42023452548). Out of 1,250 articles, 11 met the inclusion criteria. The quality of eligible articles was assessed using the Newcastle-Ottawa Scale (NOS) tool. The systematic review followed the PRISMA guidelines.

**Results:**

The serotoninergic system, particularly the 5-HTTLPR polymorphism, is consistently linked to altered connectivity in the ventral attention network, impaired inhibitory control, and increased susceptibility to AN. The 5-HTTLPR polymorphism affects reward processing, motivation, reasoning, working memory, inhibition, and outcome prediction in patients with AN. The dopaminergic system, involving genes like *COMT*, *DRD2*, *DRD3*, and *DAT1*, regulates reward, motivation, and decision-making. Genetic variations in these dopaminergic genes are associated with psychological manifestations and clinical severity in patients with AN. Across populations, the Val66Met polymorphism in the *BDNF* gene influences personality traits, eating behaviors, and emotional responses. Genes like *OXTR*, *TFAP2B*, and *KCTD15* are linked to social cognition, emotional processing, body image concerns, and personality dimensions in patients with AN.

**Conclusion:**

There was an association linking multiple genes to the susceptibly and/or severity of AN. This genetic factor contributes to the complexity of AN and leads to higher diversity of its clinical presentation. Therefore, conducting more extensive research to elucidate the underlying mechanisms of anorexia nervosa pathology is imperative for advancing our understanding and potentially developing targeted therapeutic interventions for the disorder.

**Systematic review registration**: [https://clinicaltrials.gov/], identifier [CRD42023452548].

## 1 Introduction

Anorexia nervosa (AN) is a complex neuropsychiatric disorder characterized by restricted caloric intake, resulting in dangerously low body weight, distorted body image, and an intense fear of weight gain (American Psychiatric Association, 2013). AN is a common eating disorder among female adolescents, with prevalence rates of 0.3–1.2% (Schwartz et al., 2008) and a female-to-male ratio of 10:1 (Smink et al., 2012). This gender disparity is most pronounced in female adolescents aged 15–19 years, where incidence rates are highest (Auger et al., 2023). The reasons for this gender imbalance are not fully understood, but they may involve a combination of biological, psychological, and sociocultural factors (Boerner et al., 2004; Crisp and Collaborators, 2006; Stanford and Lemberg, 2012). For instance, societal pressures and ideals regarding female body image, as well as differences in stress response and coping mechanisms between genders, may contribute to the higher prevalence in females (Crisp and Collaborators, 2006). Despite being less common in males, AN in men is often underdiagnosed and understudied, leading to a lack of awareness and understanding of the disorder in this population (Raevuori et al., 2014).

Additionally, AN is particularly serious due to the high rate of relapse (Klump et al., 2009), its medical complications resulting from severe malnutrition, as well as the elevated risk of suicidality (Attia, 2010). In 20–30% of cases, AN develops into a chronic illness persisting for many years and often over the lifetime of an individual, impairing social interaction and diminishing academic and professional potential. Notably, compared to age and sex-matched controls, people with AN have mortality rates that are five to ten times higher (Kirk et al., 2017). The chronic nature of the disease, coupled with a high relapse rate and significant personal, familial, and societal burdens, underscores the pressing need for increased research to understand its etiology and, therefore, improve treatment approaches.

Typically, patients with AN are assessed and diagnosed according to The Diagnostic and Statistical Manual of Mental Disorders, fifth edition (DSM-5) tool (American Psychiatric Association, 2013), which is the 2013 publication of the American Psychology Association (APA). A range of adverse outcomes are associated with AN, including medical and psychiatric comorbidities (Herzog et al., 1997; Fairburn and Harrison, 2003; Treasure et al., 2010), and is defined as the psychiatric disorder with the highest mortality rate (Arcelus et al., 2011; Zipfel et al., 2014). Anxiety and depression are prevalent comorbidities in individuals with AN that further exacerbate the severity and complexity of the disorder (Pleplé et al., 2021; Calvo-Rivera et al., 2022). In addition, individuals with AN tend to display personality traits characterized by a low impulsivity (Favaro et al., 2004; Testa et al., 2022), high harm avoidance (Frank et al., 2018), and perfectionism (Lloyd et al., 2014), suggesting a neurobiological basis for the disorder rooted in heightened inhibition and control mechanisms that govern behavior, emotion, and physiology (Ellison and Foong, 1998; Bulik et al., 2010; Wonderlich et al., 2018). Individuals with AN may have impairments in this inhibitory function, as seen in their struggles with set-shifting tasks that require redirecting attention away from previously relevant stimuli (Roberts et al., 2007). Psychological assessment tools such as semi-structured interviews and self-reporting questionnaires are pivotal in providing insights into diagnosis, disease severity, and comorbidities and understanding the various psychological traits and behavioral aspects of eating disorders like AN, hence the impact on the quality of life of those individuals (Schaefer et al., 2021; Wade and Pellizzer, 2024). While to understand the underlying structural and functional changes in the brain associated with AN, neuroimaging tools like Magnetic Resonance Imaging (MRI), functional Magnetic Resonance Imaging (fMRI), and Resting-state functional magnetic resonance imaging (rs-fMRI) were used. These tools detect changes in brain activity, blood flow, and oxygenation, revealing functional connectivity patterns and baseline brain activity in which variability in connectivity may be linked to cognitive abilities and personality traits (van den Eynde et al., 2012; Zhu et al., 2012). Positron Emission Tomography (PET) and Single Photon Emission Computed Tomography (SPECT) scans analyze metabolic and blood flow activity, aiding in the assessment of functional abnormalities (Blake et al., 2003). Brain research using these techniques has provided abundant evidence of neurobiological changes in eating disorders (ED).

The underlying mechanisms of the development and progression of AN are not yet fully understood. Present research indicates that the disease lies at an intersection of psychological characteristics and environmental and sociocultural factors, in addition to genetic and biological predispositions (Mazzeo and Bulik, 2009; Campbell and Peebles, 2014; Baker et al., 2017). Interestingly, family (Strober et al., 2000; Frisch et al., 2001) and twin (Klump et al., 2001; Bulik et al., 2010) studies strongly suggest a genetic component to the disorder by consistently showing that first-degree relatives of individuals with AN have an increased risk of developing the disorder compared with relatives of unaffected individuals (Bulik et al., 2007). This is reflected in the heritability rates of AN, which are estimated to be approximately 33–84% (Donato et al., 2022), indicating a significant genetic contribution to the disorder. Genetic polymorphisms, which are variations in DNA sequences among individuals, have emerged as potential candidates influencing susceptibility to the onset, progress, and development of AN (Paolacci et al., 2020). Recent advances in molecular genetics have enabled the identification of genetic polymorphisms that may confer susceptibility to AN. Variations in genes related to serotonergic, dopaminergic, opioid, and appetite regulation systems have gained particular attention for their involvement in mood and body weight regulation (Compan, 2021), reward and emotional behavior (Weiss et al., 1988; Hasan and Hunaid, 2011), as well as energy intake pathways (Monteleone et al., 2005; Perello and Dickson, 2015).

The intricate relationship between genetic polymorphisms and the traits found in AN is a critical area of investigation. Neurotransmitter imbalances, altered brain structure, and disrupted neural circuits contribute to the AN pathophysiology. The impact of hormones and neuroactive peptides, such as sex hormones and gut hormones, on brain responses disrupts normal food reward circuits (Monteleone and Maj, 2013). Structural brain alterations involve cerebral atrophy, enlarged ventricles, and changes in grey and white matter volumes (van den Eynde et al., 2012; Seitz et al., 2016). Studies examining gray matter volumes in AN demonstrate fluctuations across various brain regions, with some areas exhibiting smaller volumes, particularly linked to the severity of the illness (Martin Monzon et al., 2017). Similarly, research on white matter volume and integrity yields inconsistent findings, but alterations in structural connectivity patterns suggest compensatory changes during the recovery process (Frank et al., 2013). Functional and effective connectivity studies point to disruptions within networks associated with the executive function (Lee et al., 2014), reward processing (Cha et al., 2016), and perception (Fonville et al., 2014), potentially contributing to the altered eating behaviors seen in AN. Body image distortion, a hallmark of AN, implicates parietal and frontal regions in encoding motivational relevance to sensory events (Wagner et al., 2003) as well as prefrontal and cingulate brain response (Xu et al., 2017). The exploration of genetic polymorphisms and their correlation with neurobiological and psychological factors in AN represents a crucial step toward unraveling the intricate complexities of this debilitating disorder.

This research investigates how specific genetic polymorphisms might amplify individuals’ susceptibility to these psychological triggers, leading to the manifestation of AN. By examining the intricate gene–environment interplay, this study aims to provide a more nuanced understanding of the factors contributing to the disorder’s heterogeneity. This systematic review synthesizes evidence from diverse studies to assess and investigate the association of gene polymorphisms with psychological and neurobiological factors in patients with AN.

## 2 Materials and methods

The International Prospective Registry of Systematic Reviews (PROSPERO CRD42023452548)^1^ received and approved a copy of the review protocol. The guidelines of the Preferred Reporting of Systematic Reviews and Meta-Analyses (PRISMA) statement served as the basis for this systematic review (Liberati et al., 2009; Moher et al., 2009).

### 2.1 Search strategy

A comprehensive and systematic search was conducted across scientific databases PubMed, PsycINFO, Scopus, and Web of Science with additional hand search for relevant articles that were searched using the following search strategy: [(“anorexia nervosa” OR “eating disorders” OR “anorexia” OR “eating behavior”) AND (“genetics” OR “polymorphism” OR “genetic variation” OR “genetic factors”) AND (“brain” OR “neuroimaging” OR “neurobiology” OR “neurophysiology” OR “neurotransmitters”) AND (“psychology” OR “personality” OR “cognition” OR “emotion” OR “behavior”)]. The search was restricted to publications between 1990 and August 2023 to provide insight into the most recent developments in research within the field. Only English articles were considered for inclusion. Additionally, the reference lists of identified articles and relevant reviews were hand-searched to ensure the inclusiveness of the retrieved literature.

### 2.2 Eligibility criteria

As recommended in the PRISMA statement, eligibility was based on the PICOS criteria: population, interventions, comparison, outcome and study design (Moher et al., 2009). Through our systematic review, we will try to answer the following PICO question: Among individuals diagnosed with anorexia nervosa (P), does the presence of specific genetic polymorphisms (I) correlate with variations in neurobiological and psychological factors (O), influencing the onset, development, or severity of the disorder, as compared to those without these polymorphisms (C)?

#### 2.2.1 Population

Studies that included individuals with a confirmed diagnosis of AN, based on established diagnostic criteria such as the Diagnostic and Statistical Manual of Mental Disorders, 4th or 5th edition (DSM-4/5), were included. Studies had to exclude any participant with any past or current other medical condition, especially neuropsychiatric disorder that could misinterpreted as part of AN comorbidities. As this review focused on isolating the specific influence of genetic polymorphisms on AN and its associated neurobiological and psychological factors, included participants with other medical conditions (e.g., gastrointestinal disorders, endocrine disorders, chronic pain) or neuropsychiatric disorders (e.g., mood disorders, OCD, ADHD) could introduce confounding factors. These conditions might share symptoms with AN, potentially obscuring the true association between genetic polymorphisms and AN itself. By excluding participants with comorbidities, the review aims to achieve a clearer understanding of the independent genetic effects on AN development. Moreover, studies with an exclusive focus on populations other than individuals diagnosed with AN [e.g., Bulimia nervosa (BN), binge-eating disorder (BED), or any other disorder] were excluded.

#### 2.2.2 Intervention

In order to be included, studies had to examine the presence of specific genetic polymorphisms in the AN case group through genotyping.

#### 2.2.3 Comparison

As a comparison group, studies with a control group in which participants to be genotyped were healthy with no current or history of any ED were included. Healthy controls had to have a BMI of 18 and higher. They had to have no past or current metabolic, endocrine, or gastrointestinal illness that could affect their weight, nor should they suffer from any neuropsychiatric disorder. Studies with within-subject comparisons, i.e., where genotyped patients with AN, BN, or BED were compared, were also included. In addition, studies that only investigated one group of AN and compared those who harbor the studied SNP and those who do not are also included.

#### 2.2.4 Outcome

Studies were considered to be eligible if they included at least one variation in neurobiological and/or psychological factors influencing the onset, development, or severity of AN, such as brain imaging data (MRI, fMRI), neuroendocrine markers, neurotransmitter systems, neuropsychological assessments, or psychological traits and behaviors related to AN.

#### 2.2.5 Study design

Observational studies (case–control studies, cohort studies, and cross-sectional) investigating the association between specific genetic polymorphisms and neurobiological and/or psychological factors in individuals diagnosed with AN were considered for inclusion. Review articles, meta-analyses, systematic reviews, commentary papers, and editorials were all excluded to avoid duplication of information. Animal Studies conducted solely on animal models were excluded, as the focus is on human populations. Non-peer-reviewed sources such as conference abstracts, posters, and non-peer-reviewed publications were all excluded to ensure the quality and reliability of the included studies.

### 2.3 Study selection

**Figure 1:**
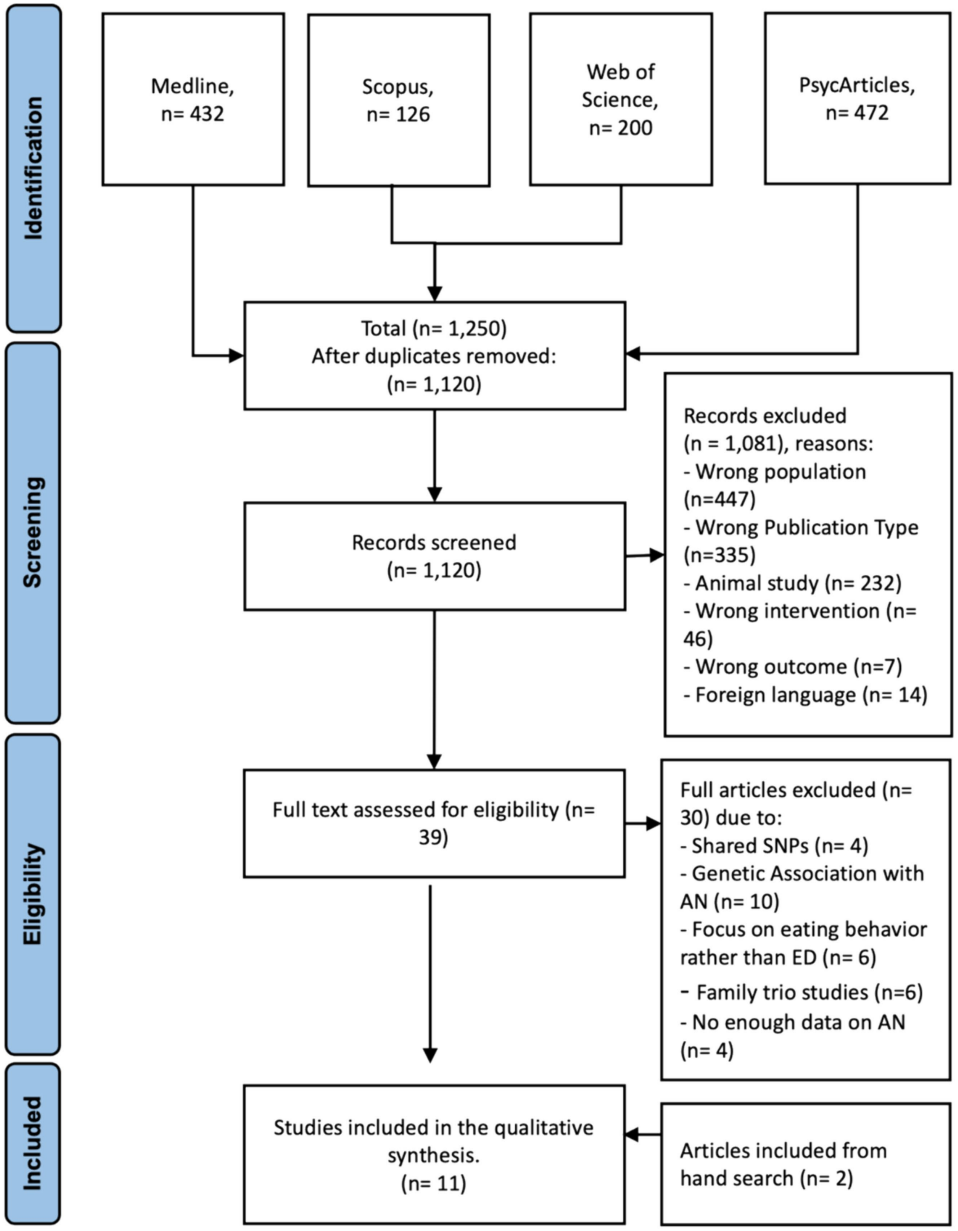
PRISMA flow chart of article selected. n = number of articles.

Initial screening of titles and abstracts was conducted by two independent reviewers (HA and HB), followed by full-text assessments of potentially eligible articles. Discrepancies were resolved through consensus. For data screening and filtration, Rayyan,^2^ an open-source website tool designed to assist researchers in systematic reviews and other knowledge synthesis initiatives, was utilized. After eliminating duplicate articles, titles and abstracts were evaluated using Rayyan, and articles were categorized as included, excluded, or maybe. Any records not meeting our inclusion criteria were excluded. During the secondary selection process, the full text of all articles was evaluated to address those initially categorized as “maybe” and finally included the eligible articles. In cases where the two reviewers disagreed on an article classified as “maybe” during the final review, discussion ensued until a consensus was reached. A PRISMA flowchart depicting the screening and selection procedure is presented in Figure 1.

### 2.4 Data extraction

For articles included in this systematic review, HA and HB collected the data independently and then discussed the final extraction. If any important data was not understood, researchers of the meant report were contacted. Variables extracted are displayed in Table 1. The following variables were extracted: (1) First Author and Year of publication; (2) study design (i.e., cross-sectional, case–control, or cohort); (3) characteristics of the study participants (sample size, gender distribution, and ethnicity); (4) Genetic polymorphisms studied (gene associated, variant investigated, and SNP ID) and finally, (5) summary of main findings as well as the associated *p* value were extracted.

**Table 1:** Summary of the main characteristics and main findings of the included studies.

References	Study design	Study participants			Neurobiological and/or psychological assessment tools		Genetic polymorphisms studied			Findings
	Study type	Cases/Controls	Ethnicity	% females	Neurobiological	Psychological	Gene	Variant/Allele	SNP ID	
Collantoni et al. (2016)	Cross-sectional	35/34	Caucasian	100%	Resting-state fMRI imaging	Stop signal task	*COMT* *SLC6A4*	Val158Met 5-HTTLPR S/L 5-HTTLPR A/G	rs25531	AN had longer SST reaction times compared to HC (*p =* 0.018). The S allele mediates the negative correlation between the positive functional connectivity of the ventral attention network and the SST reaction time (*p =* 0.043).
Clarke et al. (2016)	Cross-sectional	71/20	Caucasian	100%	Skin conductive response	Body shape questionnaire (BSQ), categorization task, and appraisal task.	*BDNF*	Val66Met	rs6265	SCR within patients was more frequent during processing of underweight stimuli compared with normal-and overweight stimuli (*p* = 0.007). The Met allele of the *BDNF* gene was associated to an increased frequency of SCR in response to cues for starvation (*p =* 0.008).
Rybakowski et al. (2007)	Case–control	149/100	Caucasian	100%	NA	Temperament and character inventory	*BDNF*	Val66Met -270C/T	rs6265	Patients with AN with Met allele showed higher harm avoidance than Val/Val homozygotes (*p =* 0.03) Patients with AN, who carried the T allele of *BDNF* –270C/T polymorphism showed higher Persistence than C/C homozygotes (*P =* <0.05). Patients with AN, carriers of the T allele of *BDNF* –270C/T polymorphism showed higher harm avoidance than C/C homozygotes (*p =* 0.006).
Kim et al. (2009)	Case–control	62/131	Korean	100%	NA	Korean version of EDE, temperament and character inventory, Y-BOCS, beck depression inventory, state and trait anxiety inventory.	*TPH1*	A218C (A/A)	_	No differences in the *TPH1* A218C allele/genotype frequency between the HC and the AN groups (*p =* 0.800). A218C allele is not associated with the psychopathologies or obsessionality of patients with AN (*p =* 0.46).
Favaro et al. (2013)	Cross-sectional	33/30	Caucasian	100%	Resting-state fMRI imaging to measure prefrontal functional connectivity	Wisconsin card sorting test, Edinburgh handedness inventory	*COMT*	Val158Met	–	Met-homozygous patients with AN showed greater coactivation in connectivity of the prefrontal cortex than Val carriers (*P =* <0.05). Patients with AN with an increasing number of Met alleles were positively associated with set-shifting impairments (*p =* 0.001)
Ando et al. (2012)	Case–control	689/573	Japanese	100%	NA	Japanese version of temperament and character inventory	*BDNF*	Val66Met	rs6265	Patients with AN carriers of Met66 allele carriers were having lower Harm avoidance scores (*p =* 0.007)
Sala et al. (2018)	Cross-sectional	49/ -	NR	100%	fMRI to measure neural responses to social stimuli	Quick inventory of depressive symptomatology, SIGH-A, YBC-EDS, Y-BOCS, the 26-item eating attitudes test, BSQ	*OXTR*	G/A	rs2254298 rs53576	Carriers of the A allele for *OXTR* rs2254298 showed reduced neural activation in response to social stimuli in all of: Medial prefrontal cortex (*p =* 0.008), Dorsal anterior cingulate (*p =* 0.02), Posterior cingulate cortex (*p =* 0.003), and Precuneus (*p =* 0.02).
Gamero-Villarroel et al. (2017)	Cross-sectional	106/ 181	Caucasian	100%	NA	Eating Disorders Inventory, and the anxiety subscale of the Symptom Checklist 90 Revised	*TFAP2B* *KCTD15*	A/G C/T G/A C/T	rs552393576 rs2817420 rs4805059 rs4239577	Carriers of A allele for *TFAP2B* rs552393576 showed significant association with the scores of drive for thinness (*p =* 0.004). Carriers of C allele for *KCTD15* rs4239577 showed significant association with the scores of ineffectiveness (*p =* 0.006). Carriers of C allele for *KCTD15* rs4239577 showed significant association with the scores of asceticism (*p =* 0.004).
Genis-Mendoza et al. (2019)	Case–control	30/292	Mexican	Cases: 76% HC: 49.7%	NA	MINI Kid, Questionnaire on Eating and Weight Pattern-Revised	*5-HTR2A*	A1438G	rs6311 rs6313	The frequency of the G allele in rs6311 SNP is higher in AN than in HC groups (*p =* 2.23e-16). Individuals with GG genotype in rs6311 SNP had an increased risk of suicide attempts (*p =* 0.035).
González et al. (2021)	Cross-sectional	210/–	Caucasian	100%	NA	Eating Disorders Inventory, and the anxiety subscale of the Symptom Checklist 90 Revised	*DRD2* *DRD3* *DAT1*	A2/A1 Ser9Gly 10R/9R	rs1800497 rs6280 rs28363170	A1/A1 carriers in the *DRD2* gene, the Gly9Gly genotype in the *DRD3* gene, and 9R/9R carriers in the *DAT1* genes all are associated with a more severe symptomology predisposition (*P =* <0.05).
Chen et al. (2015)	Case–control	255/351	Han Chinese	Cases: 95% HC: 97%	NA	EDE	*SLC6A4*	5-HTTLPR S/L	–	5-HTTLPR showed significant association with AN (*p =* 0.03). The frequency of S allele higher in AN than in HC (*p =* 0.017).

AN, Anorexia Nervosa; HC, Healthy Controls; SST, Stop Signal Task; SCR, Skin Conductance Response; EDE, Eating Disorders Examination Interview; Y-BOCS, Yale-Brown Obsessive Compulsive Scale; YBC-EDS, The Yale Brown Cornell Eating Disorder Survey; SIGH-A, The Structured Interview Guide for the Hamilton Anxiety Scale; MINI Kid, Mini International Neuropsychiatric Interview for Children and Adolescent; (−) was typed when the paper has not mentioned the intended information.

### 2.5 Quality assessment

Two researchers (HA and HB) evaluated the quality of eligible articles for risk of bias of included cross-sectional and case–control studies using the Newcastle-Ottawa Scale (NOS) tool (Stang, 2010). When using the NOS tool, three domains of scoring criteria, totaling seven in all, were applied to the included cross-sectional and case–control studies. Study quality was determined by the studies’ selection of study groups, generalizability and verification of exposure and results, with little differences in scoring parameters when adapted to different study designs and a total of eight to nine points could be earned. For cross-sectional studies, the classification of quality was as follows: studies receiving more than seven points were categorized as very good, those earning 6–5 points were considered good quality, studies obtaining 4–3 points were deemed satisfactory, and those with 2 or fewer points were labeled as poor or unsatisfactory quality. For case–control studies, the quality categorization was as follows: studies accruing more than eight points were classified as very good, those receiving 7–6 points were termed good quality, studies earning 5–4 points were seen as satisfactory, and those with 3 or fewer points were designated as poor or unsatisfactory quality. Any instances of conflicts or disagreements arising during the evaluation process were resolved through consensus discussions between the researchers.

### 2.6 Ethical considerations

All included studies were scrutinized for evidence of ethical approval and participant consent. All the studies explicitly reported obtaining formal written informed consent from participants. This review is confined to the analysis of published data, and no primary data collection from human participants was conducted as part of this study.

### 2.7 Data synthesis

Considering the heterogeneity in study designs, the genetic polymorphisms investigated, neurobiological outcome measures, and psychological factors assessed, a narrative synthesis approach was utilized. The findings of the studies were systematically summarized and organized according to the impact of genetic polymorphisms on neurobiological and psychological factors in AN.

## 3 Results and discussion

### 3.1 Search results and study selection

An initial search through Medline, Scopus, Web of Science, and PsycArticles as of 20 August 2023 yielded 1,250 studies. Following the removal of 130 duplicates, the titles and abstracts of the remaining 1,120 articles were screened. Thirty-nine articles were identified for full paper review and were assessed for eligibility; of these, 30 articles were further excluded for not fulfilling one or more of the eligibility criteria. The reasons for exclusion were the investigation of different shared SNPs between AN and other psychiatric disorders or other EDs rather than isolated investigation on AN-associated SNPs (*n* = 4), studies that did not include the investigation of identified SNPs with neither neurobiological nor psychological factors, but only the genetic association and predisposition to AN (*n* = 9), studies focused on eating behaviors without explicitly focusing on AN (*n* = 6), family trio studies (*n* = 6) since we focused on population-based studies to enhance the generalization, a lack of enough data describing AN participants (*n* = 6). Further hand search, which involved reviewing the reference lists of the articles identified through our initial database search as well as relevant review articles and meta-analyses in the field of AN and genetic polymorphisms, resulted in two additional articles (Chen et al., 2015; González et al., 2021), increasing the total included to 11. The PRISMA flowchart in Figure 1 presents the detailed selection procedure flow.

### 3.2 Quality assessment of eligible studies

Six of the eleven articles included were cross-sectional studies (Favaro et al., 2013; Clarke et al., 2016; Collantoni et al., 2016; Gamero-Villarroel et al., 2017; Sala et al., 2018; González et al., 2021), whereas the remaining five were case–control studies (Rybakowski et al., 2007; Kim et al., 2009; Ando et al., 2012; Chen et al., 2015; Genis-Mendoza et al., 2019), all of which were assessed in terms of quality using the Newcastle-Ottawa Scale (NOS) Quality Assessment criteria (Supplementary Figure S1) (Stang, 2010). None of the included studies was below the satisfactory level; in fact, nine included articles achieved a good quality level and above after the assessment (Kim et al., 2009; Ando et al., 2012; Favaro et al., 2013; Scott-van Zeeland et al., 2014; Chen et al., 2015; Clarke et al., 2016; Collantoni et al., 2016; Gamero-Villarroel et al., 2017; Genis-Mendoza et al., 2019; González et al., 2021; Supplementary Table S1).

The final quality score for the six cross-sectional studies (Favaro et al., 2013; Clarke et al., 2016; Collantoni et al., 2016; Gamero-Villarroel et al., 2017; Sala et al., 2018; González et al., 2021) that were evaluated for selection, comparability, and outcome were mostly positive—with the majority rated as “Very good” (score of 7 out of 10) for four studies (Favaro et al., 2013; Clarke et al., 2016; Collantoni et al., 2016; Gamero-Villarroel et al., 2017). One study received a “Good” rating (score of 6 out of 10) (González et al., 2021), and another one study was considered “Satisfactory” (score of 4 out of 10) (Sala et al., 2018). At a similar rate, the final quality scores for the six case–control studies that were evaluated for selection, comparability and exposure were found to meet or exceed satisfactory standards, with two studies rated “Very good” with a score of 8 (Chen et al., 2015; Genis-Mendoza et al., 2019), two studies rated “good” with scores 6 and 7 (Kim et al., 2009; Ando et al., 2012) and one study deemed “satisfactory” with a score of 5 (Rybakowski et al., 2007).

### 3.3 Characteristics of the included studies

The main characteristics of the included articles are displayed in Table 1. All 11 articles were based on subjects presenting with AN (Rybakowski et al., 2007; Kim et al., 2009; Ando et al., 2012; Favaro et al., 2013; Chen et al., 2015; Clarke et al., 2016; Collantoni et al., 2016; Gamero-Villarroel et al., 2017; Sala et al., 2018; Genis-Mendoza et al., 2019; González et al., 2021). All articles included outcomes related to relationship investigation between specific studied SNPs of genes to neurobiological and/or psychological factors to AN (Rybakowski et al., 2007; Kim et al., 2009; Ando et al., 2012; Favaro et al., 2013; Chen et al., 2015; Clarke et al., 2016; Collantoni et al., 2016; Gamero-Villarroel et al., 2017; Sala et al., 2018; Genis-Mendoza et al., 2019; González et al., 2021). All the referenced studies in this review were published between 2007 and 2021 (Rybakowski et al., 2007; Kim et al., 2009; Ando et al., 2012; Favaro et al., 2013; Scott-van Zeeland et al., 2014; Chen et al., 2015; Clarke et al., 2016; Collantoni et al., 2016; Gamero-Villarroel et al., 2017; Sala et al., 2018; Genis-Mendoza et al., 2019; Boehm et al., 2020; González et al., 2021), with a concentration of publications in the latter half of the 2010s (Ando et al., 2012; Favaro et al., 2013; Chen et al., 2015; Clarke et al., 2016; Collantoni et al., 2016; Gamero-Villarroel et al., 2017; Sala et al., 2018; Genis-Mendoza et al., 2019). The participants were either healthy individuals or had the specific condition of interest (AN) typically including underweight patients who were diagnosed according to DSM criteria. The majority of the articles had the participant diagnosis based on the DSM-4 criteria (Rybakowski et al., 2007; Kim et al., 2009; Ando et al., 2012; Favaro et al., 2013; Chen et al., 2015; Collantoni et al., 2016; Sala et al., 2018), with a few re-evaluating to comply with the DSM-5 edition (Gamero-Villarroel et al., 2017; González et al., 2021), while others directly used the DSM-5 criteria for diagnosis (Clarke et al., 2016; Genis-Mendoza et al., 2019).

Those with other significant medical conditions, psychiatric comorbidities (except anxiety), and specific medication use were excluded. Healthy Controls (HC) were generally required to have no EDs with exclusions for significant medical or psychiatric conditions or substance abuse. All of the studies had all-female participants with the exception of two studies that had mixed-gender participants (Chen et al., 2015; Genis-Mendoza et al., 2019), with females comprising the majority of the group. The ethnic composition of the study participants was diverse, with six studies involving Caucasian participants (Rybakowski et al., 2007; Favaro et al., 2013; Clarke et al., 2016; Collantoni et al., 2016; Gamero-Villarroel et al., 2017; González et al., 2021), one study involving Mexican participants (Genis-Mendoza et al., 2019), one involving Japanese participants (Ando et al., 2012), one involving Korean participants (Kim et al., 2009), one involving Han Chinese participants (Chen et al., 2015), and one study not specifying the ethnicity but mentioned that the participants were recruited from Texas in the United States (Sala et al., 2018), while another was conducted in Germany without specifying the recruitment source (Boehm et al., 2020). The sample size in these studies varied widely, ranging from smaller groups of 49 AN cases to large cohorts of 689 AN cases, often paired with a comparable number of HC. The age range and mean ages of AN and HC participants across the studies spanned from early adolescence to mid-adulthood.

Notably, the results of the included studies, which involved other subgroups of different EDs, such as BN or BED, were filtered to include only results related to patients with AN in comparison to healthy controls. This helped accurately identify the possible correlation between the studied polymorphism and the neurobiological or psychological factors of AN as a unique ED.

### 3.4 Genetic polymorphism characteristics

The genetic polymorphisms investigated in these 11 studies focused on the polymorphisms within 1 or 2 target genes. The most common gene evaluated was the *BDNF* gene (Rybakowski et al., 2007; Ando et al., 2012; Clarke et al., 2016), followed by the *SLC6A4* (Chen et al., 2015; Collantoni et al., 2016) and the *COMT* gene (Favaro et al., 2013; Collantoni et al., 2016), which were the genes of interest in 2 studies each. The rest of the genes *TPH1* (Kim et al., 2009), *OXTR* (Sala et al., 2018), *KCTD1* (Gamero-Villarroel et al., 2017), *TFAP2B* (Gamero-Villarroel et al., 2017), *5HTR2A* (Genis-Mendoza et al., 2019), *DRD2* (González et al., 2021), *DRD3* (González et al., 2021) and *DAT1* (González et al., 2021) were evaluated only in 1 study each. Among these 11, three studies evaluated the association of genetic polymorphism with both neurobiological factors and psychological factors (Favaro et al., 2013; Collantoni et al., 2016; Sala et al., 2018), whereas the other 8 studies evaluated the association with only psychological factors (Rybakowski et al., 2007; Kim et al., 2009; Ando et al., 2012; Chen et al., 2015; Clarke et al., 2016; Gamero-Villarroel et al., 2017; Genis-Mendoza et al., 2019; González et al., 2021). All the three studies that looked at neurobiological implications in the AN individuals employed neuroimaging techniques such as fMRI (Favaro et al., 2013; Collantoni et al., 2016; Sala et al., 2018). The correlation between the studied factors and the investigated genetic polymorphism was then reported in a separate column as studies’ findings.

### 3.5 Association of serotonergic system-related gene polymorphisms with psychological and neurobiological factors in patients with AN

**Figure 2:**
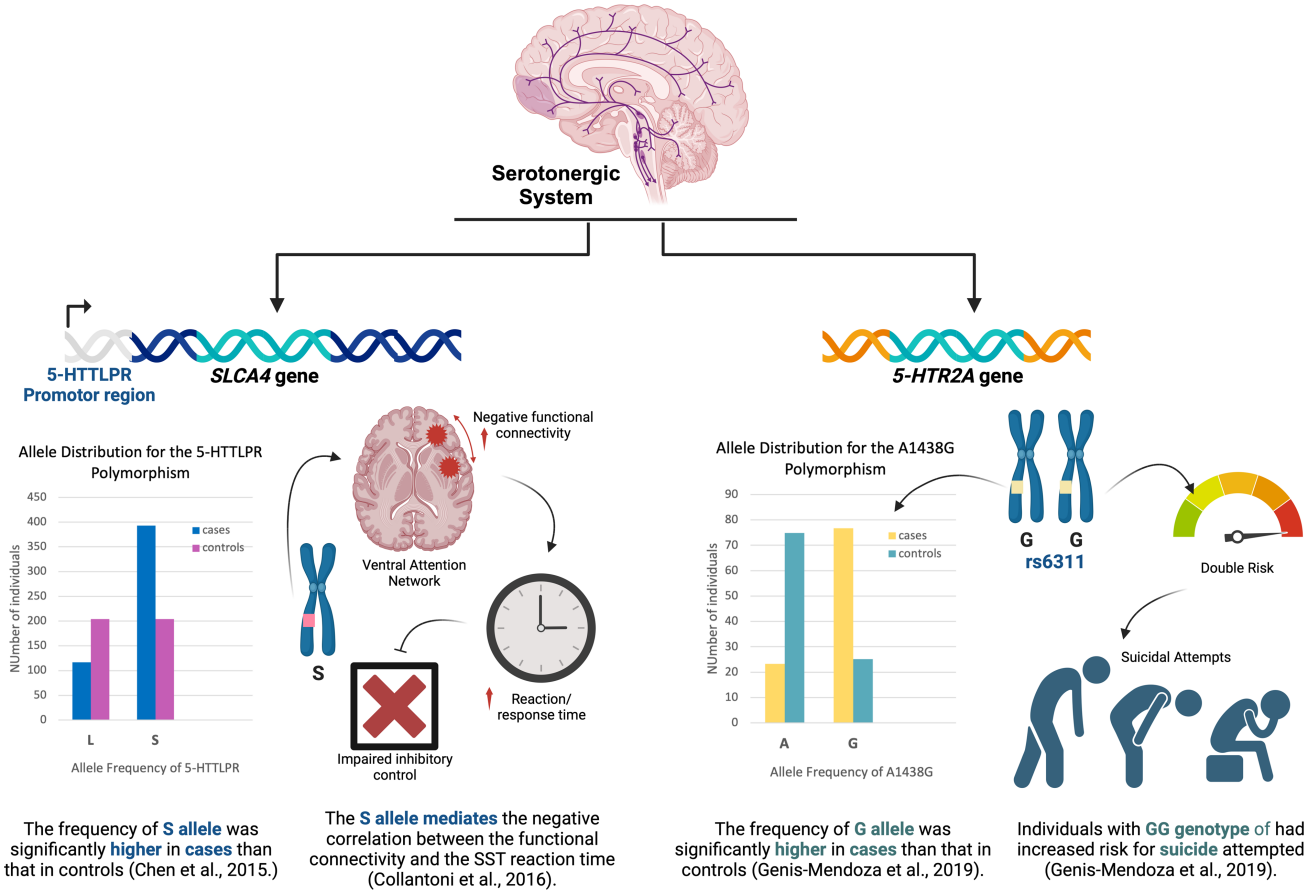
Key findings of the studies that explored the serotonergic system.

Serotonin dysfunction has been shown to have an involvement in almost all neuropsychiatric conditions (Marazziti, 2017), including EDs (Pugsley et al., 1978; Steiger, 2004). Its role in the regulation of eating behavior has been well-confirmed (van Galen et al., 2021). Polymorphisms in the serotonin-related genes have been shown to influence the psychopathological features of EDs (Gervasini et al., 2012). Across the studies included in this review, four studies investigated the association of genetic polymorphisms in the serotonin-related genes including *SLC6A4* (Chen et al., 2015; Collantoni et al., 2016), *TPH1*, and *5-HT2R* with the psychological profiles and/or neurobiological characteristics of patients with AN. Figure 2 highlights the most important findings of the included studies which investigated the serotonergic system. The *SLC6A4* gene codes for the serotonin transporter protein that carries serotonin from the synaptic cleft back into the presynaptic neuron (Mia et al., 2022). The degenerate repeat sequence in the promoter region of this gene, called the 5HTTLPR (5HT Transporter Linked Promoter Region), occurs in two common polymorphs designated as the short “S” and the long “L” alleles (Heils et al., 1996). Two of the reviewed studies investigated the polymorphism in the 5HTTLPR region of this gene (Chen et al., 2015; Collantoni et al., 2016). The *TPH1* gene codes for the Tryptophan hydroxylase enzyme that catalyzes the rate-limiting step of serotonin synthesis. *TPH1* is expressed in the body but not the brain (Walther et al., 2003). Nevertheless, the effect of variations in the *TPH1* gene on brain-related variables, such as personality traits and neuropsychiatric disorders, has been studied (New et al., 1998; Allen et al., 2008). The *5HTR2A* gene belongs to the G-protein coupled serotonin receptor family and codes for a Gq/G11-coupled receptor, which mainly plays a role in neuronal excitation in the central nervous system (Beliveau et al., 2017).

Collantoni et al. (2016) suggested that there is a correlation between the functional connectivity within the ventral attention network and reaction times measured by the stop signal task (SST), and that this relationship is mediated by the presence of the short allele (S allele) of the 5-HTTLPR polymorphism, proposing by that a potential modulatory role of the serotoninergic system in the ventral attention network. In patients with AN, positive connectivity in the ventral attention circuit correlated with response times in carriers of the LL genotype, while the opposite was true in carriers of the S allele. In other words, for patients with AN S allele carriers, negative connectivity is associated with longer response times suggesting impaired inhibitory control. This subsequently correlates with the severity of the disorder since there was a significant negative correlation between task performance and the lowest lifetime BMI. Ultimately, these correlations may reflect alterations in the mechanisms that regulate goal-oriented attention and self-referential processes in patients with AN (McFadden et al., 2014). This effect on the ventral attention network suggests that genetic variation can disrupt the processing reward, motivation, reasoning, working memory, inhibition, as well as outcome prediction (Krawczyk, 2002). In addition, the modulatory effect of the 5-HTTLPR polymorphism on the connectivity of the ventral attention network can also explain the impaired response inhibition in patients with AN as this network has been implicated in stimulus-driven attentional control (Corbetta and Shulman, 2002). Meanwhile, Chen et al. (2015) study looked into this gene polymorphism in Han Chinese patients with AN for association with psychological traits. These traits were measured using the Eating Disorders Examination (EDE) Interview as the assessment tool. They found that the frequency of the S allele of the 5-HTTLPR region of the SCL6A gene was significantly higher in patients than in controls, hence, associating with increased predisposition to AN. However, they did not find any clinical correlation or significant association between this genetic polymorphism and the severity of AN psychological symptoms. The same was reported by a meta-analysis done in 2015. Based on their results, the S allele was found to be significantly more prevalent in the AN group than in the control group (*p* = 0.006). Moreover, upon ethnically stratified analysis, it was observed that only Caucasians showed a correlation between the 5-HTTLPR polymorphism and AN, while no such correlation was found in Asians (Chen and Qian, 2015). Another meta-analysis of 15 studies concluded that being a carrier of the 5-HTTLPR S allele represents a risk factor for eating disorders (Solmi et al., 2016).

Historically, in 1996, it was discovered that the human serotonin transporter gene (SLC6A4; also known as 5-HTT) has a repeat length polymorphism in its promoter region, which regulates gene expression *in vitro*. People with one or two copies of the S allele of 5-HTTLPR have been found to exhibit elevated neuroticism, a personality trait that is linked to depression (Lesch et al., 1996). In 2002, it was reported that individuals carrying the S allele show increased amygdala reactivity to threatening stimuli, as determined by functional MRI (Hariri et al., 2002). In 2003, it was reported that S-carriers are more likely to experience depressive symptoms, have diagnosable depression, and exhibit suicidal tendencies after experiencing stressful life events and childhood maltreatment (Caspi et al., 2003). From there, a study has suggested that carriers of the S allele tend to exhibit lower expression of the serotonin transporter (SLC6A4), resulting in reduced reuptake of serotonin (5-HT) from the synapse. As a result, this reduced reuptake may lead to stronger psychopathological reactions to stressful experiences in individuals with the S allele compared to those with the L allele (Li, 2006).

In sum, the collected evidence strongly suggests that 5-HTTLPR polymorphism, particularly the S allele, is associated with altered functional connectivity in the ventral attention network, impaired inhibitory control, and increased predisposition to AN. However, a meta-analysis that included biobank data analysis concluded no significant difference in low-functioning genotype (S allele) and allele frequencies between AN group and controls, suggesting no potential association of 5-HTTLPR polymorphism with AN (Solmi et al., 2016).

Another study by Genis-Mendoza et al. (2019) in the Mexican population investigated two different SNPs (rs6311 and rs6313) in the *5HTR2A* gene. They used two different psychological assessment tools, the Mini International Neuropsychiatric Interview for Children and Adolescents (MINI Kid) and Questionnaire on Eating and Weight Pattern (QEWP), to assess different comorbidities associated with AN disorder and other EDs, including major depressive episodes, suicidality, dysthymia disorder, attention-deficit/hyperactivity disorder, generalized anxiety disorder, oppositional defiant disorder, and psychotic disorder. The results were different between the two SNPs investigated. The G allele of rs6311 (−1438G/A) was much more common in the AN group and increased AN risk by almost nine-fold, as it was hypothesized that carriers of the G allele are susceptible to express the behavioral traits before the disease onset. It was also shown that rs6311 SNP is associated with an increased risk of suicide risk. No positive associations were observed for rs6313 SNP.

A study by Kim et al. (2009) looked at the *TPH1* gene variation in a group of Korean AN patients. They investigated the genetic variant A218C (rs1800532) in the *TPH1* gene among patients with AN and HC. Using the Korean version of Eating Disorder Examination (EDE), Temperament and Character Inventory (TCI), Yale-Brown Obsessive Compulsive Scale (Y-BOCS), and State–Trait Anxiety Inventory (STAI), they assessed the psychological factors of restraint, eating concern, weight concern, shape concern subscales, persistence and harm avoidance, obsessive-compulsive symptoms, as well as the level of depression and anxiety in patients with AN and HC. Interestingly, there were no differences in the *TPH1* A218C allele/genotype frequency between the healthy controls and the individuals with AN. In addition, although the A218C variant in the TPH1 gene is reported to be related to a higher risk of suicidal behavior (Franken et al., 2005) and acute depression (Ikemoto and Panksepp, 1999), it was found by Kim et al. not to be associated with psychopathologies or obsessionally of patients with AN. Thus far, one could speculate that the *SCL6A4* and the *5HTR2A* may have a more direct and potent impact on serotonin reuptake. This could influence behaviors associated with AN more significantly than the *TPH1* gene, which is involved in the earlier stages of serotonin synthesis and might be buffered by compensatory mechanisms.

### 3.6 Association of dopaminergic system-related gene polymorphisms with psychological and neurobiological factors in patients with AN

Dopamine has also long been proposed to play a central part in the pathophysiology symptoms observed in individuals with AN. Dopamine regulates the reward system (Lippa et al., 1973; Wise, 1978), specifically the mood (Ashby and Isen, 1999), feeding behavior (Szczypka et al., 1999), motivation (Blackburn et al., 1992), and decision-making (Assadi et al., 2009). Disruption of the brain reward system is a common observation in individuals with AN (Avena and Bocarsly, 2012). It has been proved that dopamine-related genes have been shown to influence the psychopathological features of EDs (Gervasini et al., 2013). Dopamine is thought to play a role in the development of AN by promoting reward and motivation (Franken et al., 2005). Higher levels of dopamine may make individuals with AN more susceptible to engaging in reward-seeking behaviors, such as restricting food intake (Ikemoto and Panksepp, 1999). A recent perspective article hypothesizes a two-stage role of dopamine in AN. In the first stage, when AN develops, dieting and exercise increase dopamine activity, which helps lock in the habits of severe dieting and weight loss. In the second stage, when these behaviors become deeply ingrained, an ongoing lack of food causes dopamine activity to drop as part of the body’s response to long-term stress (Beeler and Burghardt, 2022).

**Figure 3:**
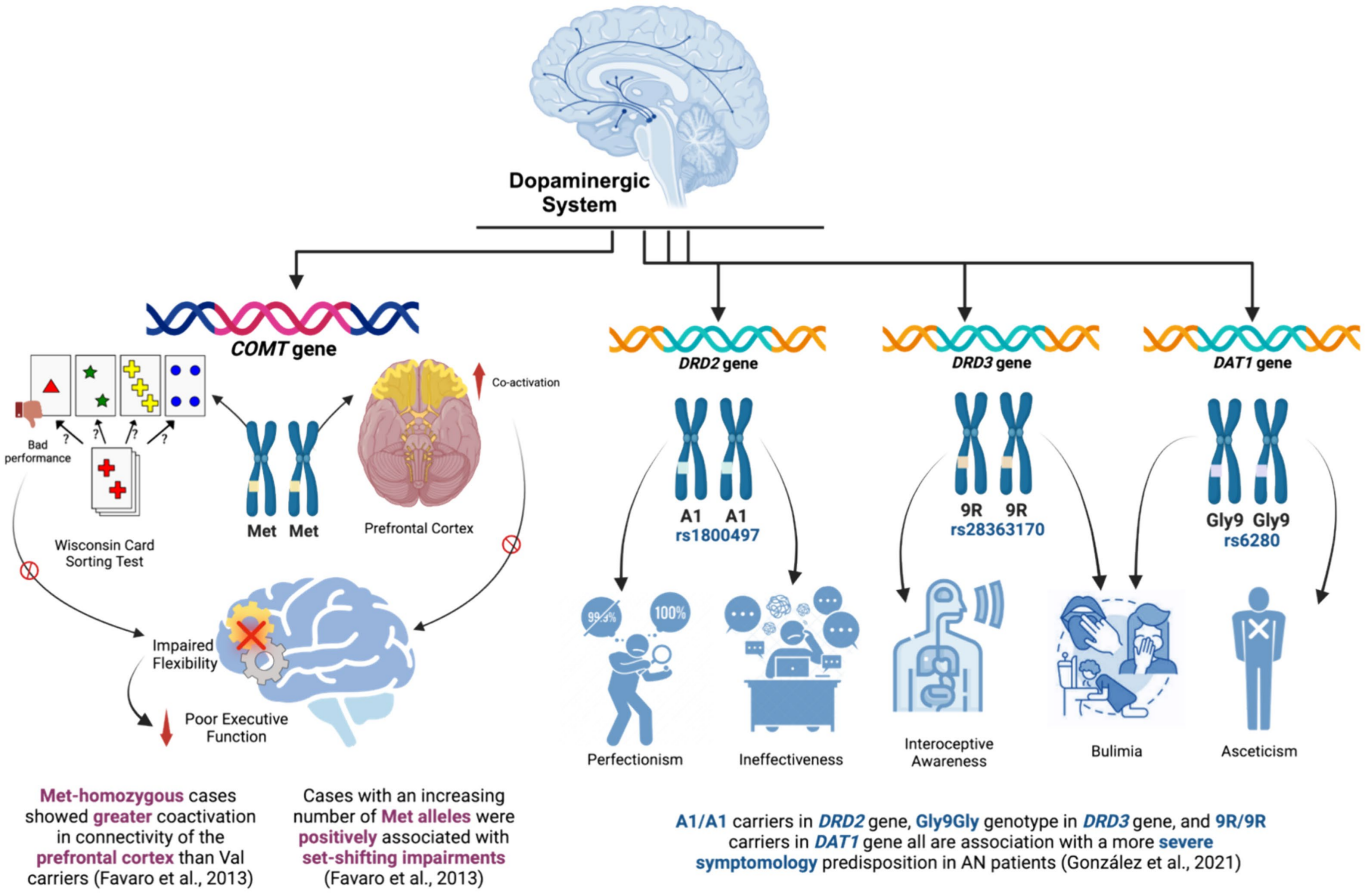
Key findings of the studies that explored the dopaminergic system.

In the present systematic review, three studies investigated the association of genetic polymorphisms in the dopamine-related genes, including *COMT* (Favaro et al., 2013; Collantoni et al., 2016) as well as *DRD2, DRD3*, and *DAT1 (*González et al., 2021) with the psychological profiles and/or neurobiological characteristics of patients with AN. Figure 3 presents the key findings of the studies that explored the dopaminergic system. The *COMT* gene codes for catechol-O-methyltransferase, an enzyme that plays a role in the breakdown of catecholamine neurotransmitters such as dopamine, norepinephrine, and epinephrine (Qayyum et al., 2015), hence regulating synaptic dopamine (Malhotra et al., 2002). *DRD2* and *DRD3* code for 2 subtypes of Dopamine receptors, coding for D2 and D3 subtypes, respectively. They have been found to regulate the synthesis, storage, and release of dopamine (Cooper, 2011). Whereas the *DAT1* gene (also known as *SLC6A3)* codes for dopamine transporter, an integral membrane protein that reuptakes and transports the dopamine from the synaptic cleft into the cytosol of surrounding cells (Baeuchl et al., 2019). Together, these three genes are an essential part of the dopamine pathway.

Firstly, the *COMT* gene was evaluated by two studies (Favaro et al., 2013; Collantoni et al., 2016) to investigate its association with the neurological factors of patients with AN. Favaro et al. (2013) studied the impact of the functional polymorphism Val158Met in the *COMT* gene on set-shifting abilities and prefrontal functional connectivity in patients with AN. The study provided evidence that the Val158Met *COMT* polymorphism has an effect on prefrontal cortex functional connectivity in patients with AN. In other words, Met-homozygous patients showed greater coactivation in the dorsolateral and ventromedial prefrontal cortex (PFC) compared to Val carriers. These brain areas relate to cognitive functions like conflict and error monitoring, reward-based learning, and decision-making (Nejati et al., 2021). Moreover, patients with an increasing number of Met alleles were positively associated with bad performance in the Wisconsin Card Sorting Test (WCST), which assesses abstract reasoning and set-shifting abilities and is considered a measure of executive functions (Kohli and Kaur, 2006). Hence, the study suggests that the studied genetic polymorphism in the *COMT* gene is associated with poor executive performance, in terms of inflexibility, and PFC functional connectivity in patients with AN. However, in a later study by Collantoni et al. (2016) SST psychological tool was used to assess the emotional response inhibition, behavioral inhibition, cognitive flexibility, and response monitoring in patients with AN. Interestingly, when investigating significant associations between SST reaction times and connectivity of the right inferior gyrus according to the presence of the Val158Met variant of the *COMT* genotype, no significant correlation was found.

Executive functions are cognitive processes that support goal-directed behavior (Luciana and Nelson, 2001). These processes are orchestrated by activity within the prefrontal cortex (Shimamura, 2000), including inhibition (behavioral and interference control), working memory, decision-making, problem-solving, and cognitive flexibility (mental flexibility or set shifting, closely associated with creativity) (Miyake et al., 2000; Lehto et al., 2003). In a recent separate study, it was confirmed that executive function impairment in ED is significantly associated with more severe symptoms and may result in a negative treatment outcome (Diaz-Marsa et al., 2023). This conclusion was reached after observing that patients with ED had significantly lower scores than HC in the performance of several executive function tests, particularly in the domains of set-shifting, interference control, and processing speed (Diaz-Marsa et al., 2023). According to the included study published by Favaro et al. (2013), patients with AN with Met homozygosity have poor set-shifting, leading to worse WCST performance than HC. The study also showed abnormal regional cortical processing in the prefrontal cortex area of the brain, which is responsible for executive functions, including cognitive flexibility and the other aforementioned processes. This can be explained by the effect of the Val158Met variant, a Val allele associated with a high-activity state of COMT, which increases the rate of dopamine degradation. This leads to lower levels of dopamine in PFC. On the other hand, the Met allele produces COMT with a low activity state, which slows the rate of degradation, resulting in higher levels of dopamine. Due to the crucial role that PFC dopamine plays in set-shifting and attention, these genetic-related differences in levels of PFC dopamine were found to be associated with different performances in executive function tasks (Esmaiel et al., 2020). Notably, elevated dopamine levels in the prefrontal cortex have been associated with impaired cognitive function mediated by this brain region since 1996 (Murphy et al., 1996). Contradictory to that conclusion, a systematic review done in 2020 suggested no association between the *COMT* gene and AN susceptibility. They suggested that the inconsistency observed could be due to the presence of other SNPs that are also in linkage disequilibrium or SNPs present in genes other than the COMT. These SNPs may have a controlling effect on the association of COMT Val158Met with AN patients (Abou Al Hassan et al., 2021).

Moreover, a large Spanish study by González et al. (2021) investigated genetic polymorphisms in 3 genes related to the dopaminergic system. This study evaluated the genetic polymorphisms of A2/A1 (rs1800497), Ser9Gly (rs6280) and 10R/9R (rs28363170) in the DRD2, DRD3, and DAT1 genes, respectively. Eating Disorder Inventory-2 (EDI-2) and the Symptom Checklist-90-Revised (SCL90R) were used as psychological assessment tools to investigate several AN-related symptoms, such as drive for thinness, bulimia, body dissatisfaction, ineffectiveness, perfectionism, interpersonal distrust, interoceptive awareness, maturity fears, asceticism, impulse regulation, and social insecurity, with and without the aforementioned genetic variations in *DRD2*, *DRD3*, and *DAT1* genes. The study found that patients with AN who carried the Gly9Gly genotype in the dopamine D3 receptor had an overall significantly worse symptomatology than those with other genotypes. In essence, those patients had far higher EDI-2 scores than the rest of the patients did (relating to bulimia and interoceptive awareness as AN-related symptoms). Whereas for *DAT1* gene, *DAT1* 9R/9R carriers had higher scores for both bulimia and asceticism compared with 10R/10R-10R/9R carriers. Elevated scores for perfectionism and ineffectiveness were displayed in *DRD2* A1/A1 carriers. Taken together, this study indicates that these specific genetic variations in the dopaminergic *DRD2*, *DRD3*, and *DAT1* genes have illustrated significant association with the predisposition with a more severe clinical picture of psychological manifestations presented in patients with AN.

In the past years, different polymorphisms of the DRD2, DRD3, and DAT1 genes appear to play a role in the vulnerability to AN (Gervasini et al., 2013). In 2005, a research paper concluded that the D2 receptor gene is a susceptibility factor in the development of AN, establishing a foundational link between genetic variations in DRD2 and EDs (Bergen et al., 2005). Beyond AN, *DRD2* was also linked to an increased risk of developing pathological eating behavior (Nisoli et al., 2007). In fact, previous studies suggested that homozygous A1/A1 subjects possess a lower number of DRD2 in brain areas (Thompson et al., 1997). The A1 allele of DRD2 has been linked not only to perfectionism and ineffectiveness, but also to the dysregulation of the body’s primary stress systems, namely the hypothalamic–pituitary–adrenal (HPA) axis (Belda and Armario, 2009), which predisposes to worse AN symptoms. Furthermore, researchers have noted a specific correlation between self-oriented perfectionism and EDs, distinguishing it from correlations with depression or adaptive disorders (Castro-Fornieles et al., 2007). Regarding the *DRD3* gene, our included study suggests a significant effect of the Gly9Gy genotype on worsened AN symptoms predisposition, especially for bulimia and interoceptive awareness (González et al., 2021). This can correlate to the DRD3 gene association with emotional and food motivational responses (Sokoloff et al., 1990), given that D3 receptor is expressed in brain regions thought to govern emotion and emotional responses to stress, reward motivation, and executive function (Sokoloff et al., 1990). Notably, the *DRD3* gene was found to play a role in the etiology of a wide range of psychopathologies, including obsessive-compulsive personality disorder (Light et al., 2006), which was found to be a very common comorbidity of AN (Serpell et al., 2002). On the other hand, the *DAT1* gene is associated with higher scores for asceticism in patients with AN (González et al., 2021). Asceticism is the practice of self-denial, often for religious or moral reasons. Individuals with AN may engage in asceticism as a way to control their weight or to express their distress. Interestingly, one paper found that as BMI decreases, religious favor of asceticism increases (Smith et al., 2004). Suggesting a potential link between asceticism and the drive for thinness, which is one of the most common motivational cues leading to EDs (Chernyak and Lowe, 2010).

### 3.7 Association of other gene polymorphisms with psychological and/or neurobiological factors in patients with AN

**Figure 4:**
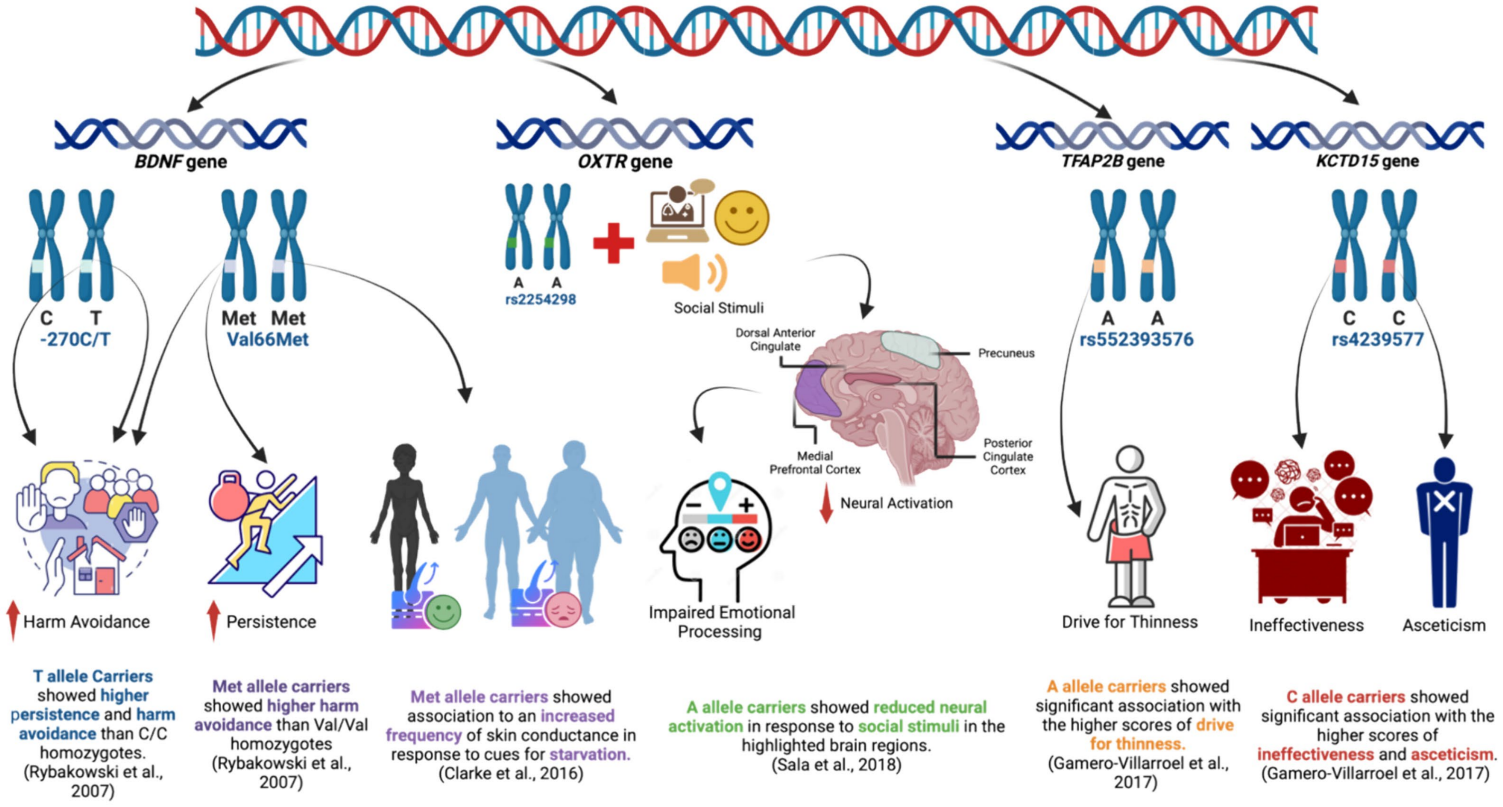
Key findings on the association of *BNDF, OXTR, TFAP2B*, and *KCTD15* gene polymorphisms with psychological and/or neurobiological factors in patients with AN.

In this systematic review, five included studies investigated the association of genetic polymorphisms in several other genes, apart from these monoamine neurotransmitter genes, with the psychological profiles and/or neurobiological characteristics of patients with AN. Out of these 3 studies investigated the *BDNF* gene polymorphism (Rybakowski et al., 2007; Ando et al., 2012; Clarke et al., 2016), one study looked into the *OXTR* gene (Sala et al., 2018), and another study focused on the polymorphisms within two genes, *KCTD1* and *TFAP2B* (Gamero-Villarroel et al., 2017). Figure 4 illustrates the main findings of this section.

BDNF gene plays a crucial role in regulating neurotransmitter systems and has been of interest in the field of EDs (Hashimoto et al., 2005; Nakazato et al., 2012). The *BDNF* gene codes for the brain-derived neurotrophic factor (BDNF), a significant growth factor belonging to the neurotrophin family (Nagahara and Tuszynski, 2011). It greatly impacts the growth and differentiation of cells and also influences the plasticity of synapses and the survival of neurons (Bathina and Das, 2015). BDNF is released in varying amounts from neurons in different regions of the brain, such as the hippocampus and ventral tegmental area (Kowiański et al., 2018). These regions are known to play a vital role in learning and reward processes (Homan et al., 2014). In individuals with AN, these processes are believed to be affected (Wierenga et al., 2022). BDNF neurons in the anterior paraventricular nucleus of the hypothalamus suppress hunger and food intake while promoting physical activity (An et al., 2015). Therefore, there is substantial evidence that supports the interaction between BDNF and eating behavior, as well as the regulation of body weight. Interestingly, The Val66Met rs6265 in the *BDNF* gene is a widely researched polymorphism in brain function, mental health, and a plethora of mental disorders (Shen et al., 2018; Wang et al., 2023).

Included in our systematic review is one of the earliest studies by Rybakowski et al. (2007), where they analyzed two polymorphisms in the *BDNF* gene, the Val66Met and-270C/T using the Temperament and Character Inventory (TCI) tool to assess different personality dimensions including novelty-seeking, harm avoidance, reward dependence, persistence, self-directedness, cooperativeness, self-transcendence in AN and HC (Aluja et al., 2010). They found that the Val166Met was associated with harm avoidance observed in patients with AN while the-270C/T was associated with both higher persistence and harm avoidance. However, both were not directly associated with AN diagnosis from the psychological point of view; rather, both were found to predispose to higher risk affecting specific personality traits, including higher persistence and harm avoidance. Harm avoidance is characterized by excessive worrying, pessimism, shyness, and being fearful, suspicious, and easily fatigued (Karppanen et al., 2023), while persistence is the tendency to continue working on something notwithstanding fatigue or frustration (Constantin et al., 2012), which are both common observations patients with AN.

The variant Val66Met of the BDNF gene was also investigated by Ando et al. (2012) using the same psychological tool used by Rybakowski et al. (2007), TCI, with some modifications that would suit the Japanese population. Their findings were contrary to Rybakowski’s in terms of having lower harm avoidance scores among Met66 carriers with no other association with reward-seeking, reward dependence, and persistence between Met66 carriers and non-carriers patients with AN. This was supported by a family trio study, which suggested that BDNF Val66Met polymorphism may not have shown a significant association with AN (Dardennes et al., 2007).

Most recently, Clarke et al. (2016) investigated the correlation between a specific SNP and psychological traits in individuals with AN. The study utilized multiple psychological assessment tools, including the skin conductance response, which is a psychophysiological measure related to the autonomic nervous system (ANS) that is responsible for various involuntary bodily functions related to stress and arousal (Bradley et al., 2001). The results showed that individuals with AN had a higher frequency of skin conductance response when exposed to images of underweight bodies. This suggests that underweight body images had a more pronounced emotional impact on individuals with AN. The study also explored the association between the Val66Met polymorphism and the frequency of skin conductance response during the processing of underweight stimuli. The findings revealed a significant association between skin conductance response frequency and the presence of the *BDNF* Met genotype. In addition, the Body Shape Questionnaire (BSQ) was also used, which is a self-report tool that assesses body image concerns and dissatisfaction (Yurtsever et al., 2022). According to the study, individuals with AN experienced more positive feelings when processing images of underweight bodies (Clarke et al., 2016), suggesting that underweight stimuli evoked a distinct emotional reaction in patients with AN and could be related to their body image perception. On the other hand, patients with AN experienced more negative feelings when processing images of normal-weight and overweight bodies, indicating that individuals with AN may have heightened negative emotional responses to images of bodies within the normal or higher weight range (Clarke et al., 2016). These findings strongly support the distorted body image hallmark of patients with AN and suggest that the Val66Met polymorphism might play a role in mediating the heightened reward value associated with starvation imagery in AN.

In conclusion, these three aforementioned studies demonstrate that the BDNF gene, especially the Val66Met polymorphism, appears to be implicated in the personality traits, eating behaviors, and emotional responses associated with AN in different populations. Consistent with those findings, in a recent study conducted by Abou Al Hassan et al. (2021), a meta-analysis was performed to examine the influence of the BDNF gene on the progression of AN. Among various factors analyzed, the researchers found evidence suggesting a connection between the restrictive subtype of AN and the phenotypic distribution of the Val66Met gene polymorphism within the *BDNF* gene. Additionally, the presence of the Met66-allele was linked to typical clinical characteristics observed in individuals with AN (Abou Al Hassan et al., 2021). Animal studies on the same variant change were also shown to promote anorectic behavior in mice, when food restriction was paired with stress such as social isolation, but only in the peri-pubertal period (Madra and Zeltser, 2016).

Another gene studied in relation to AN is the *OXTR* gene, which codes for the oxytocin receptor protein. The role of the neuropeptide oxytocin (OT) in facilitating a range of social processes is well established (MacDonald and MacDonald, 2010). It was reported that *OXTR* polymorphisms are associated with social/emotional/behavioral functioning in children and adolescents (Kohlhoff et al., 2022). Sala et al. (2018), an included study in our systematic review, investigated two different SNPs in the *OXTR* gene, rs2254298 (G/A) and rs53576 (G/A), in patients with AN. They assessed the neural response to social stimuli using functional MRI in different brain regions. In contrast to previously reported data, no significant differences were found in the examined brain regions based on the rs53576 genotype. At the same time, carriers of the A allele for *OXTR* rs2254298 showed reduced neural activation in response to social stimuli in the medial prefrontal cortex, dorsal anterior cingulate, posterior cingulate cortex, and precuneus. Interestingly, the medial prefrontal cortex, dorsal anterior cingulate, posterior cingulate cortex, and precuneus are all areas associated with social cognition, emotional processing, and self-referential thinking (McAdams and Krawczyk, 2011, 2014; Schulte-Rüther et al., 2012; McAdams et al., 2015, 2016). Those processes are usually impaired in patients with AN (Oldershaw et al., 2011; Tchanturia et al., 2011; Lang et al., 2016). Consistent with this, another study showed that the A allele carriers of rs2254298 exhibited greater global social impairments (Parker et al., 2014). The study by Sala et al. (2018) further assessed its preliminary findings by the eating attitude test (EAT-26) as a psychological tool to assess the ED behaviors and ED symptomatology related to shape and weight concerns for significantly higher measures to be found for rs2254298A carriers compared to rs2244298GG carriers (Sala et al., 2018). That would suggest a potentially significant association between this genetic variation and disturbed body image and distorted perception of weight, which are central aspects of AN.

In a review of EDs and oxytocin receptor polymorphisms (Burmester et al., 2021), they observed that research on the *OXTR* SNPs rs53576 and rs2254298 has revealed distinct associations between genetic variations and eating behaviors. Specifically, the A allele of both SNPs is independently linked to restrictive eating behaviors, while the G allele of rs53576 is associated with binging behaviors, a correlation reflected in neuroanatomical findings (Micali et al., 2017). Interestingly, one study discovered that the A allele of *OXTR* SNPs poses a risk for more severe symptoms related to ED, whereas the G allele provides some protective effects (Acevedo et al., 2015). Despite these associations, individual *OXTR* SNPs alone are unlikely to fully explain the complexity of EDs, suggesting that they may influence the expression and/or effectiveness of the *OXTR*.

*TFAP2B* and *KCTD1* are two other genes investigated in an included study by Gamero-Villarroel et al. (2017). Transcription Factor AP-2 Beta (*TFAP2B*) and the Potassium Channel Tetramerization Domain Containing 15 (*KCTD15*) are two obesity-related genes that interact to regulate feeding behavior (Williams et al., 2014). *TFAP2B* is a crucial regulator of monoaminergic genes (Damberg, 2005), while *KCTD15* is reported to be associated with putative regulation of body mass index (BMI) (Mei et al., 2012). In the prespective included cross-sectional study, ten clinically relevant and tag single-nucleotide polymorphisms (SNPs) in *KCTD15* and *TFAP2B* were screened in the included participants in both AN and HC. The EDI-2 and the anxiety subscale of the Symptom Checklist 90 Revised (SCL90R) were utilized as psychological assessment tools to assess all of the drive for thinness, bulimia, body dissatisfaction, ineffectiveness, perfectionism, interpersonal distrust, interoceptive awareness, maturity fears, asceticism, impulse regulation, and social insecurity. The results showed that the effect of the gene–gene interaction was more profound in scales such as perfectionism, maturity fears, or social insecurity for patients with AN. Moreover, in terms of the correlation with the studied SNPs, rs552393576 and rs2817420 in *TFAP2B* and rs4805059 and rs4239577 in *KCTD15* showed significant relevant associations with the scores of several dimensions, including drive for thinness, ineffectiveness, and asceticism. In other words, the study suggests that genetic variability in *TFAP2B* and *KCTD15* genes may influence personality dimensions related to AN, suggesting that the interaction of genetic variability in these loci could influence the risk for ED and/or psychological parameters.

Interestingly, both *TFAP2B* and *KCTD15* genes were reported by multiple studies to be related to higher obesity risk (Bauer et al., 2009; Willer et al., 2009; Albuquerque et al., 2014; Lv et al., 2015) and there are many shared personality dimensions and risk factors between obesity and ED. These include body dissatisfaction, low self-esteem, anxiety, depression, substance abuse, dieting, binge-eating, and a history of sexual/physical abuse (Day et al., 2009). With that being said and given that both genes were found to alter personality dimensions, it is possible that genetic variations of both genes would not specifically predispose to more severe psychological manifestations of AN specifically, but rather in any eating disorder.

## 4 Conclusion

In conclusion, this comprehensive review highlights the intricate relationship between genetic polymorphisms and the psychological and neurobiological factors associated with AN. The serotoninergic system, particularly the 5-HTTLPR polymorphism, has been consistently implicated in altered functional connectivity within the ventral attention network, impaired inhibitory control, and an increased predisposition to AN. The findings emphasize the impact of the 5-HTTLPR polymorphism on the ventral attention network, shedding light on the potential mechanisms underlying altered reward processing, motivation, reasoning, working memory, inhibition, and outcome prediction in patients with AN. The dopaminergic system, encompassing genes such as *COMT*, *DRD2*, *DRD3*, and *DAT1*, plays a crucial role in regulating reward, motivation, and decision-making processes. Genetic variations in these dopaminergic genes have been linked to diverse psychological manifestations and clinical severity in AN patients.

The *BDNF* gene, specifically the Val66Met polymorphism, emerges as a significant player in influencing personality traits, eating behaviors, and emotional responses associated with AN across diverse populations. The *OXTR* gene, responsible for oxytocin receptor protein, and other genes like *TFAP2B* and *KCTD15*, reveal intriguing associations with social cognition, emotional processing, body image concerns, and personality dimensions in patients with AN. It is evident that genetic variations contribute to the complexity of AN, influencing not only the susceptibility to the disorder but also shaping its diverse clinical presentations. Future research should continue exploring the interplay between genetic factors and environmental influences to provide a more comprehensive understanding of the etiology and pathophysiology of AN. There is a need for further investigation to extend these findings to eating pathology. The use of Genome-Wide Association Studies (GWAS) could significantly contribute to expanding our knowledge in this area, shedding light on the intricate interplay between genetics and environmental factors in the development of EDs. This knowledge could pave the way for personalized therapeutic interventions that target specific genetic vulnerabilities, ultimately improving outcomes for individuals affected by this challenging and potentially life-threatening condition.

## 5 Strengths and limitations

This is the first systematic review to evaluate the genetic polymorphisms and their association with neurobiological and psychological factors in anorexia nervosa status and summarize almost all the available evidence regarding this association. In addition, a rigorous systematic approach was followed to develop this review, such as the PRISMA guidelines and the Rayyan application, which was used to perform initial screening and enabled the research team to conduct a blind review that would enhance the credibility of the findings. Additionally, the studies included were conducted on a large number of participants, who were relatively homogenous for their age (mainly adolescents) and health status (all cases were clinically diagnosed according to internationally established criteria). Additionally, the studies included in this systematic review were conducted in various countries, which allows us to see the influence of different genetic polymorphisms on neurobiological and psychological factors in individuals with various genetic backgrounds. Furthermore, the quality of the included studies was mainly high, and only two out of eleven studies were of stationary quality, which indicates that the evidence drawn from these studies is unlikely to be biased or the result of other uncontrolled confounding factors.

Despite the valuable insights provided by the reviewed studies on the association between genetic polymorphisms and psychological/neurobiological factors in AN, several limitations should be considered. Many studies exhibit small sample sizes, potentially compromising the generalizability of findings. Using cross-sectional designs might limit the ability to establish causation or determine the direction of relationships. Publication bias may skew the overall understanding, as positive results are more likely to be published. Multiple testing and the lack of replication studies pose risks of false-positive results. Psychological assessment tools vary across studies, impacting the comparability of results. The complexity of gene–gene interactions and the functional implications of genetic variations require further exploration. Addressing these limitations in future research endeavors will contribute to a more nuanced understanding of the genetic factors influencing AN.

## Data availability statement

The original contributions presented in the study are included in the article/Supplementary material, further inquiries can be directed to the corresponding author.

## Author contributions

HA: Writing – original draft, Visualization, Methodology, Investigation, Data curation, Conceptualization. HB: Writing – review & editing, Validation, Supervision.

## Footnotes

^1^ https://www.crd.york.ac.uk/prospero/

^2^ https://rayyan.ai/
