# Supplementary material for: Genetic polymorphisms and their association with neurobiological and psychological factors in anorexia nervosa: a systematic review
Source: Front Psychol. 2024 Jun 21;15:1386233. doi: 10.3389/fpsyg.2024.1386233 (PMC11229080; doi:10.3389/fpsyg.2024.1386233)
Supplement: Supplementary file 1 [file Data_Sheet_1.docx]

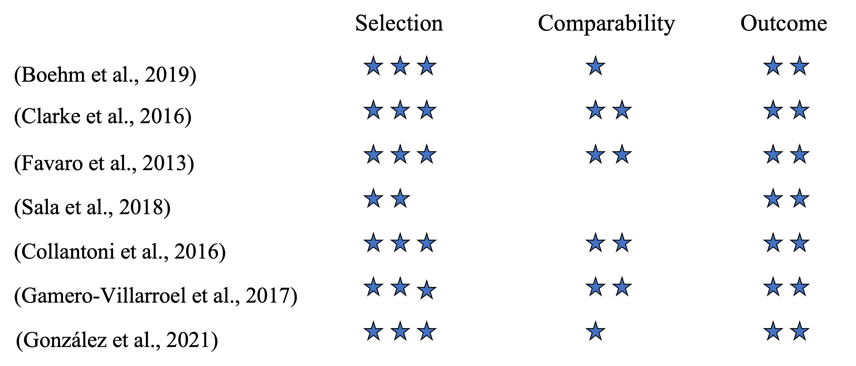


**Figure 1 Supplementary |** Total scores representing the quality of included cross-sectional studies in terms of selection, comparability, and outcome.


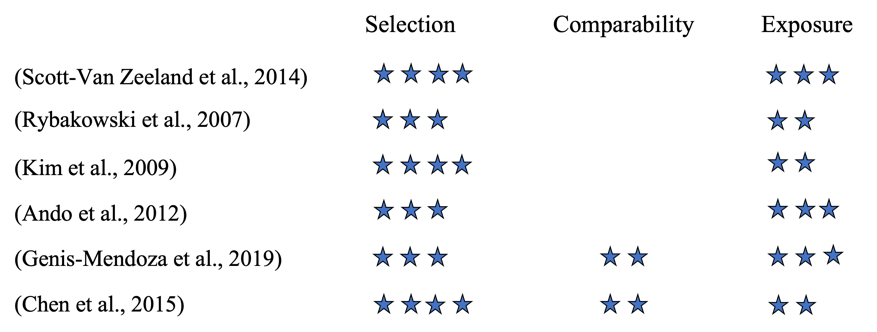


**Figure 2 Supplementary |** Total scores representing the quality of included cross-sectional studies in terms of selection, comparability, and exposure.

| Table 1 Supplementary \| Quality assessment for studies included | | | | | | | | | | | | |
| --- | --- | --- | --- | --- | --- | --- | --- | --- | --- | --- | --- | --- |
| Section A \| Newcastle-Ottawa Quality Assessment Scale criteria for cross-sectional studies | | | | | | | | | | | | |
| study | Selection | | | | | | Comparability | Outcome | | | | Final Quality score |
|  | Representativeness of the sample | | Sample size | Non-respondents | Ascertainment of the exposure | | The potential confounders were investigated | Assessment of the outcome | | Statistical test | |  |
| (Boehm et al., 2019)-[48] | * | | * | - | * | | * | * | | * | | 6- Good |
| (Clarke et al., 2016)-[49] | * | | * | - | * | | ** | * | | * | | 7 - Very Good |
| (Favaro et al., 2013) -[50] | * | | * | - | * | | ** | * | | * | | 7 - Very Good |
| (Sala et al., 2018) -[51] | 0 | | * | - | * | | - | * | | * | | 4- Satisfactory |
| (Collantoni et al., 2016) -[52] | * | | * | - | * | | ** | * | | * | | 7- Very Good |
| (Gamero-Villarroel et al., 2017) -[53] | * | | * | - | * | | ** | * | | * | | 7- Very Good |
| (González et al., 2021) -[54] | * | | * | - | * | | * | * | | * | | 6- Good |
| Section B \| Newcastle-Ottawa Quality Assessment Scale criteria for case-control studies | | | | | | | | | | | | |
| study | Selection | | | | | | Comparability | Exposure | | | | Final Quality score |
|  | Is the case definition adequate | Representativeness of the cases | | Selection of Controls | | Definition of Controls | Comparability of cases and controls on the basis of the design or analysis | Assessment of the exposure | Same method of ascertainment for cases and controls | | Non-Response rate |  |
| (Scott-Van Zeeland et al., 2014)- [55] | * | * | | * | | * | 0 | * | * | | * | 7 - Good |
| (Rybakowski et al., 2007) - [56] | * | 0 | | * | | * | 0 | * | * | | 0 | 5- Satisfactory |
| (Kim et al., 2009) -[57] | * | * | | * | | * | 0 | * | * | | 0 | 6- Good |
| (Ando et al., 2012) -[58] | * | 0 | | * | | * | 0 | * | * | | * | 6- Good |
| (Genis-Mendoza et al., 2019) -[59] | * | 0 | | * | | * | ** | * | * | | * | 8 - Very Good |
| (Chen et al., 2015) -[60] | * | * | | * | | * | ** | * | * | | 0 | 8 - Very Good |
